# The multiomic landscape of meningiomas: a review and update

**DOI:** 10.1007/s11060-023-04253-2

**Published:** 2023-02-25

**Authors:** Justin Z. Wang, Farshad Nassiri, Alexander P. Landry, Vikas Patil, Jeff Liu, Kenneth Aldape, Andrew Gao, Gelareh Zadeh

**Affiliations:** 1 MacFeeters Hamilton Neuro-Oncology Program, Princess Margaret Cancer Centre, University Health Network and University of Toronto, Toronto, ON Canada; 2 Division of Neurosurgery, Department of Surgery, University of Toronto, Toronto, ON Canada; 3 Princess Margaret Cancer Centre, University Health Network, Toronto, ON Canada; 4 The International Consortium on Meningiomas, Toronto, ON Canada; 5 Laboratory of Pathology, Center for Cancer Research, National Cancer Institute, Bethesda, MD USA; 6 Laboratory Medicine Program, University Health Network, Toronto, ON Canada

**Keywords:** Meningiomas, Molecular, Multiomics, Genomics, Epigenomics, Classification

## Abstract

**Purpose:**

Meningiomas are the most common primary brain tumor in adults. Traditionally they have been understudied compared to other central nervous system (CNS) tumors. However over the last decade, there has been renewed interest in uncovering the molecular topography of these tumors, with landmark studies identifying key driver alterations contributing to meningioma development and progression. Recent work from several independent research groups have integrated different genomic and epigenomic platforms to develop a molecular-based classification scheme for meningiomas that could supersede histopathological grading in terms of diagnostic accuracy, biological relevance, and outcome prediction, keeping pace with contemporary grading schemes for other CNS tumors including gliomas and medulloblastomas.

**Methods:**

Here we summarize the studies that have uncovered key alterations in meningiomas which builds towards the discovery of consensus molecular groups in meningiomas by integrating these findings. These groups supersede WHO grade and other clinical factors in being able to accurately predict tumor biology and clinical outcomes following surgery.

**Results:**

Despite differences in the nomenclature of recently uncovered molecular groups across different studies, the biological similarities between these groups enables us to likely reconciliate these groups into four consensus molecular groups: two benign groups largely dichotomized by *NF2*-status, and two clinically aggressive groups defined by their hypermetabolic transcriptome, and by their preponderance of proliferative, cell-cycling pathways respectively.

**Conclusion:**

Future work, including by our group and others are underway to validate these molecular groups and harmonize the nomenclature for routine clinical use.

## Introduction

Although meningiomas are the most common primary brain tumour in adults, they have been significantly understudied compared to other central nervous system (CNS) tumors [1]. Part of this is likely attributable to the idea that most of these tumours are benign and can be successfully treated with surgery and/or radiotherapy (RT) [2]. However, we now understand that a significant proportion of meningiomas (20–30%) are clinically aggressive and have a proclivity to recur with significant morbidity and even mortality. While there is no accepted definition of “poor outcome” in meningioma, aggressive subtypes often have a 5-year progression free survival (PFS) probability less than 50%. There is therefore an urgent need to develop more accurate diagnostic tools and targeted treatments for this population, which is bound to continue increasing in size as our population ages. Importantly, our classification of other CNS tumours such as gliomas relies heavily on molecular status whereas meningiomas have been almost entirely classified using histopathology until recently in 2021, when the World Health Organization (WHO) grading incorporated homozygous loss of *CDKN2A/B* (cyclin-dependent kinase inhibitor 2A/B) and *TERT* (telomerase reverse transcriptase) promoter (*TERTp*) mutation as criteria for grade 3 meningiomas.

A recent review has comprehensively outlined different prognostic alterations in meningiomas [3]. We will briefly summarize a few of these landmark studies as it relates to more recent work focused on the integration of these alterations across different genomics platforms to develop molecular classification schemes for meningiomas.

### Loss of NF2 identified in sporadic meningiomas

The first genetic alteration identified in meningiomas arose from patients with neurofibromatosis 2 (NF2), an autosomal dominant tumor syndrome caused by biallelic inactivation of the *NF2* gene, resulting in meningiomas that affect up to half of these patients in addition to pathognomonic development of bilateral vestibular schwannomas [4–7]. In 1994 Ruttledge et al. noted loss of heterozygosity (LOH) of chromosome 22 in 50% of meningiomas in their cohort of 170 tumors [8, 9]. Alterations in *NF2* remain the most common genetic abnormality in meningiomas, found in up to 60% of sporadic cases, and its inactivation has been hypothesized to be an early tumorigenic event.

### Non-NF2 mutations

In 2013, two separate landmark studies utilized whole genome and whole exome sequencing to uncover novel mutations in non-*NF2* meningiomas including *TRAF7* (TNF receptor associated factor 7), *KLF4* (Krüppel-like factor 4), *AKT1*, and *SMO* (Smoothened) [10, 11]. *TRAF7* mutations were found in approximately a quarter of all meningiomas in one study (72/300, 24%) [11, 12]. These mutations almost always co-occurred with recurrent *KLF4* mutations (K409Q) [13, 14]. The E17K variant of the *AKT1* mutation were the next most commonly detected alteration, and were found to frequently co-occur with *TRAF7* mutations (25/38, 66%) but were mutually exclusive with *KLF4* mutations [15]. Lastly *SMO* mutations were found in a minority of meningiomas without alterations in the other genes above [10, 11, 16]. Most meningiomas with these non-*NF2* mutations tended to be more benign, of lower WHO grade, and associated with less chromosomal abnormalities. [11]

### Other prognostic mutations in meningiomas

Since the discovery of non-*NF2* driver mutations in meningiomas, other prognostically important genomic alterations have since been uncovered (Table 1). *POLR2A* (RNA polymerase II polypeptide A) mutations were found more commonly in benign meningiomas without large-scale chromosomal alterations [17]. In familial studies of patients with schwannomatosis and multiple meningiomas, germline mutations in the tumor suppressor gene *SMARCB1* (SWIch/Sucrose Non-Fermentable (SWI/SNF)-related matrix-associated actin-dependent regulator of chromatin subfamily B member 1) and *NF2* were identified as the key predisposing alterations [18–21]. One of the rare but early prognostically important mutations in clinically aggressive meningiomas was the *TERTp* mutation, found to be present in 16/252 (6.4%) of meningiomas in one study and were associated with significantly shorter TTP compared to *TERT* wild-type meningiomas irrespective of WHO grade. [22, 23] *TERTp* mutations have since been added to the most recent iteration of the WHO classification of CNS tumors as an independent marker of grade 3 meningiomas, and increased sensitivity of *TERTp* mutated meningioma cells to ETS transcription factor inhibition has emerged as a potential therapeutic strategy. [24, 25]

**Table 1:** Genes implicated in meningiomas and meningioma development

Gene	Location	Gene status	Frequency	WHO grade	Modification	Effect of modification
NF2	22q12.2	Tumor suppressor	40–60%	1–3	Deletion/non-sense mutation	Loss of merlin, a cytoskeleton scaffolding protein
TRAF7	16p13.3	Tumor suppressor	20–25%	1	Missense mutation	Dysregulation of E3 ubiquitin ligase interaction with MAPK pathway
KLF4	9p31	Tumor suppressor	10–15%	1	Missense mutation	Loss of transcriptional regulation
AKT1	14q32.33	Oncogene	10%	1	Point mutation	Constitutive activation of downstream mTOR signalling
SMO	7p32.1	Oncogene	1–5%	1	Point mutation	Constitutive activation of the SHH signalling pathway
PIK3CA	3q26.32	Oncogene	5%	1	Point mutation	Constitutive activation of PI3 kinase and PI3K/AKT pathway
POLR2A	17p13.1	Oncogene	5%	1	Missense mutation	Hijacking of the catalytic subunit of RNA polymerase II
BAP1	3p21.1	Tumor suppressor	< 1%	2, 3	Splice site/non-sense/frameshift mutation	Inactivation or loss of nuclear localization of a ubiquitin carboxy-terminal hydrolase
SMARCB1	22q11.23	Tumor suppressor	< 5%	2, 3	Missense mutation	Inactivation of core subunit of SWI/SNF chromatin remodeling complex
SMARCE1	17q21.2	Tumor suppressor	< 1%	1	Splice site/non-sense/frameshift mutation	Inactivation of subunit of SWI/SNF chromatin remodeling complex
BRAF (V600E)	7p34	Oncogene	< 1%	3	Missense mutation	Increase in BRAF activity (downstream of Ras)
CHEK2	22q12.1	Tumor suppressor	50%	1–3	Deletion/frameshift mutation	Impairment of DNA repair and increased chromosomal instability
PTEN	10q23.31	Tumor suppressor	2–6%	2, 3	Frameshift mutation/deletion/rearrangement	Loss of negative regulation of AKT/PKB signalling pathway
CDKN2C	1p32.3	Tumor suppressor	1%	2	Nonsense mutation	Loss of regulation of CDK4, CDK6,
TERTp	5p15.33	Oncogene	5.5%	3*	Point mutation	Activation of telomerase-mediated telomere stabilization

*As per the 2021 WHO classification of brain tumors grading criteria

While histopathology continued to drive the contemporary grading of meningiomas, interesting corollaries between pathological subtype and molecular findings have been uncovered *SMARCE1* (SWI/SNF-related matrix-associated actin-dependent regulator of chromatin subfamily E member 1) mutations were found in almost all cases of meningiomas with clear cell histology (WHO grade 2) and are often mutually exclusively with *NF2* loss and non-*NF2* mutations [26–29]. Subsequent studies demonstrated *SMARCE1* loss to be associated with reduced DNA accessibility over distal enhancer sites, and *SMARCE1*-deficient cells were susceptible to mSWI/SNF inhibition [29]. Another rare alteration in aggressive meningiomas was inactivation of *BAP1* [breast cancer (BRCA)1-associated protein-1 tumor suppressor] [30–32]. *BAP1* loss on IHC was more common in histologically rhabdoid and papillary meningiomas (WHO grade 3), although more cases are being reported in exclusion of these histological subtypes [31]. Less than 30 cases have reported, including germline and somatic *BAP1* mutations, and almost universally with poor outcomes. [31, 32]

### Copy number alterations

Aside from chromosome 22q and the *NF2* gene, cytogenetic studies have shown meningiomas to be characterized by complex patterns of other chromosomal losses and gains that vary with biological aggressiveness (Table 2).

**Table 2:** Common and prognostic copy number alterations in meningiomas

Chromosome arm/gene	Loss/Gain	Approximate frequency in all meningiomas	Contribution to clinical outcome
1p	Loss	30–50%	Unfavourable
3q	Loss	10–15%	Unfavourable
4p	Loss	5–10%	Unfavourable
6p	Loss	10%	Unfavourable
6q	Loss	15–20%	Unfavourable
7p	Loss	5%	Unfavourable
8p	Gain	< 5%	Unknown
10q	Loss	10%	Unfavourable
11q	Loss	5%	Unfavourable
14q	Loss	20%	Unfavourable
15q	Gain	< 5%	Unknown
16q	Gain	5%	Unknown
17q	Gain	5–10%	Unknown
18q	Loss	15–20%	Unfavourable
20q	Gain	10%	Unknown
22q	Loss	50–60%	Unfavourable
CDKN2A/B	Loss (homozygous)	1–5%	Unfavourable

Deletions of the short arm of chromosome 1 (1p) have been implicated as an early step in meningioma development and a factor contributing to tumor progression [33, 34]. Loss of heterozygosity (LOH) of 1p was associated with chromosome 22/*NF2* deletions in meningiomas, and poorer PFS. In almost all meningiomas with copy number imbalances, there is associated 1p loss [35, 36]. In addition to 1p, losses of 6p, 10q, 18q, and gains of 17q and 20q were found to be recurrent across high-grade meningiomas [37–39]. In a cohort of 527 meningiomas, prognostic copy number alterations and histopathological features such as number of mitoses were integrated to develop a “molecularly integrated grade” [37, 40]. Copy number alterations most associated with shorter time to progression (TTP) in their LASSO regression model were losses of 1p, 6q, 10q, 18q, 19p, and *CDKN2A/B*. [40]

The only other molecular criteria incorporated into the 2021 WHO classification was homozygous loss of *CDKN2A/B*, adjacent tumor suppressor genes on chromosome 9p21 which inhibit cell cycle G1 progression through the inactivation of CDK4 and CDK6 [41, 42]. Sievers et al. analyzed *CDKN2A/B* homozygous deletion in 528 meningioma patients using DNA methylation and found this alteration in 26 cases (4.9%, 7 WHO grade 2 and 19 WHO grade 3) with significantly shorter TTP compared to tumors without this deletion [43]. Bi et al. corroborated these findings but did not find significant differences in outcome between tumors with homozygous versus heterozygous *CDKN2A/B* loss. [40]

### DNA methylation classification

In 2017, our group and Sahm et al. published the first studies on DNA-methylation based classification systems for meningioma, showing better predictive power for clinical outcomes and biology compared to WHO grade [44, 45]. Using genome-wide DNA methylation profiles, we applied unsupervised clustering to uncover two major epigenetic groups of meningiomas which were discernable based on a 64 CpG loci predictor, termed MM-FAV (prognostically favourable subgroup) and MM-UNFAV (prognostically unfavourable subgroup). The MM-UNFAV subgroup was associated with several CNAs including losses of 1p, 6q, 14q, and 18q and gain of 1q.

Sahm et al. also identified two methylation-driven subgroups, termed groups A and B. These were then subdivided: group A into 4 subgroups (3 benign, 1 intermediate), and B into 2 (1 intermediate and 1 malignant). These six methylation subgroups were termed MC ben-1, MC ben-2, MC ben-3, MC int-A, MC int-B, and MC mal, with the latter two classes comprising the most aggressive (and least common) meningiomas. The benign methylation classes were enriched for WHO grade 1 meningiomas, whereas the malignant class contained the majority of WHO grade 3 cases. WHO grade 2 meningiomas were largely distributed between the two intermediate classes (MC int-A and -B). The methylation groups also had a unique distribution of recurrent mutations. MC ben-1 meningiomas were enriched in *NF2* mutations while the MC ben-2 subgroup was enriched in non-*NF2* mutations including TRAF7, AKT1, KLF4, and SMO. MC ben-3, MC int-B, and MC-mal had similar proportions of *NF2*-mutant meningiomas ranging from 31 to 35% of meningiomas in each class, while 53% of meningiomas in MC int-A were *NF2*-altered. Nearly all meningiomas with *TERTp* mutations were found in the more aggressive epigenetic group B classes and 70% of all meningiomas with *CDKN2A/B* deletion belonged to the MC mal class [45]. Group B meningiomas also demonstrated a higher burden of copy number alterations including loss of 1p.

Given the highly prognostic role of DNA methylation in outcome prediction, our group leveraged these signatures to build a prognostic model of meningioma recurrence after surgery. We found that our model was independently associated with PFS after adjusting for WHO grade, extent of resection, and burden of copy number alterations. By combining this methylation signature with WHO grade and Simpson grade, we developed a clinically applicable nomogram to predict five-year recurrence risk on individual tumours, which outperforms traditional metrics such as WHO grade or Simpson grade alone. [46]

### Integration of multiple genomic platforms for classification

Until recently, there was no consensus for classifying meningiomas into meaningful biological or molecular subgroups as for medulloblastomas or gliomas [47–49]. Despite the discrepancies in classification when different genomics platforms are used, there were sufficient similarities between groups that a unified classification criteria should be viable. Table 3 details the main findings from three recent studies that have utilized multiomic methods to classify meningiomas, which we will summarize below.

**Table 3:** Molecular subgroups and descriptions of multiomic classes from different studies

Study	Derivation of molecular group	Group	WHO Grades	Mutations	CNV	Transcriptional profile	Clinical outcome
Nassiri et al.	Unsupervised sample-wise consensus hierarchical clustering of gene level somatic copy number alterations, DNA methylome, and transcriptome data followed by COCA	MG1: Immunogenic	1	*NF2*	22q-	Immune regulation and signaling pathways	Good
		MG2: NF2-wildtype	1, 2	*TRAF7, AKT1, KLF4, POLR2A*	5p/q+, 12p/q+, 13p/q+, 17p/q+,20p/q+	Vasculature and angiogenic pathways	Intermediate
		MG3: Hypermetabolic	2, 1	*NF2, DMD, TERTp, CHD2, CREBBP*	1p−, 6q−, 14p/q−, 22q−	Metabolism of macromolecules	Poor
		MG4: Proliferative	2, 3	*NF2, TERTp, KDM6A, CHD2, CREBBP, PTEN, RB1, FBXW7*	1p−, 1q+, 6p/q−, 10p/q−, 14p/q−, 22q−, *CDKN2A/B-*	Cell cycle regulation, proliferation associated transcription factor networks (MYC, FOXM1, E2F)	Poor
Bayley et al.	NMF and k-means clustering based on most variably methylated CpGs, followed by PLS models with DNA methylation and CNV data as independent data, and RNA-seq as the dependent data	MenG A	1	*TRAF7, KLF4, AKT1, PI3KCA, SMO, POLR2A, TERTp*	None	Vasculature development and cell cycle signaling	Good
		MenG B	2, 1	*NF2, SMARCB1*	22q-	Immune signaling and Hedgehog pathway	Good
		MenG C	2, 1	*NF2, ARID1A, TERTp*	1p-, 22q-	Metabolic and cell cycle pathways	Poor
Choudhury et al.	PCA of most variable probes in DNA methylation data followed by K-means consensus clustering, continuous distribution functions, and unsupervised hierarchical clustering	Merlin-intact	1	*TRAF7, AKT1, KLF4*	5p+, 6p-	*NF2* expression	Good
		Immune-enriched	1, 2	*NF2*	6p+, 22q-	HLA and meningeal lymphatic genes	Intermediate
		Hypermitotic*	2, 1, 3	*NF2*	1p−/+, 6p−, 9p−, 14q−/+, 22q−, *CDKN2A/B*−	FOXM1 and other cell cycle pathways	Poor

*COCA* cluster of cluster analysis; *NMF* non-negative matrix factorization; *PLS* partial least squares; *PCA* principal component analysis

*Subsequently found to contain two separate methylation subgroups, corresponding to hypermetabolic and proliferative subgroups with distinct clinical outcomes (Choudhury et al.)

In 2021, we published on a comprehensive, integrative analysis of matched whole genome molecular data that included DNA somatic copy number, point mutations, DNA methylation, mRNA expression, whole cell proteomics, and single cell RNA sequencing on the same meningiomas. We found that these different platforms provided complementary and non-redundant information at the gene level. Instead of using a single clustering method on a single type of genomics data, we utilized a multilayered cluster of cluster algorithm to uncover 4 stable molecular groups, which we abbreviated MG 1-4. Though this initial clustering process was agnostic to clinical data, meningiomas belonging to each of these MGs had distinctly different clinical outcomes with progressively shorter TTP with increasing MG. Classification by MG was superior to WHO grade, previous methylation-only classification, and classification by individual genomic datatypes in predicting outcome. The mutational profile of each group was similarly unique. Nearly all MG1 meningiomas had *NF2* mutations whereas almost no MG2 meningiomas had this alteration. MG2 meningiomas were instead enriched for non-*NF2* mutations *AKT1*, *TRAF7*, *KLF4*, and *POLR2A*. We also identified novel somatic driver mutations in our aggressive MG3 and MG4 meningiomas, including chromatin remodeling and epigenetic regulators *KDM6A*, *CHD2*, and the tumor suppressor *PTEN*. Our most aggressive group, MG4 had the highest mutational burden compared to all other MG. On a copy number level, MG1 meningiomas were relatively bereft of significant alterations save for loss of chromosome 22q. While most MG2 meningiomas were copy number neutral, polysomies of chromosomes 5, 12, 13, 17, and 20 were seen for meningiomas with angiomatous and microcystic histology [37, 39]. In contrast, MG3 and MG4 meningiomas harboured a higher number of copy number alterations, specifically losses of 22q, 1p, 6q, 14p/q, and 18p/q. Each MG also had its own unique transcriptomic profile. MG1 meningiomas were enriched for pathways involved in immune signaling and regulation, therefore we termed this group “immunogenic”. MG2 meningiomas, primarily “NF2-wildtype”, had transcriptomic pathways involved in vasculature development and angiogenesis. MG3 meningiomas were enriched for pathways that involved metabolism of different macromolecules, giving this group its “hypermetabolic” name. Lastly, MG4 meningiomas, consisting of the most aggressive and “proliferative” meningiomas, were enriched for cell cycle regulation pathways including *MYC*, *FOXM1, E2F*, etc. Using single cell RNAseq (scRNAseq), we found limited intratumoral heterogeneity in most meningiomas, with most neoplastic cells of a given patient’s tumor resembling the molecular signatures of the bulk tumor. Furthermore, deconvolution of bulk mRNA data using neoplastic and non-neoplastic signatures from our scRNAseq data demonstrated a high level of concordance with our MG. Bulk proteomics data corroborated these transcriptomic pathways within each MG and highly abundant protein targets with significant IHC correlates were identified in for each MG. However, these specific IHC stains could be utilized to identify MG independent of sequencing and molecular stratification on a 1:1 basis, requires additional validation. [50]

Following our study, Bayley et al. performed initial DNA methylation-based clustering of 110 primary meningiomas (WHO grades 1 and 2), and found 3 stable methylation clusters [45, 51]. They then classified these same meningiomas based on transcriptomic profile (RNAseq), copy number status, and *NF2* status/degree of chromosomal instability, then assessed the degree of agreement between classification using these 4 different platforms. They found that 100/110 (91%) fit into 3 groups based on agreement of at least 3/4 genomics platforms. These groups were termed MenG A, B, and C. MenG A meningiomas were almost entirely WHO grade 1, had no cytogenetic changes, and were *NF2*-wildtype. MenG B meningiomas were *NF2*-deficient with 22q loss, but had a low degree of chromosomal instability, and could not be distinguished from MenG A based on clinical outcome. MenG C meningiomas were *NF2*-deficient with associated 1p loss in addition to having a higher burden of other copy number alterations, and clinically were the poorest performers. Interestingly MenG C meningiomas were also the most heterogeneous tumors as transcriptional profiling had the lowest degree of specificity in differentiating MenG C meningiomas from the other groups. [51]

Nearly concurrently with the above study, Choudhury et al. published on the classification of their cohort of meningiomas driven by DNA methylation. Using multiple different approaches, they were able to reproducibly generate 3 meningioma DNA methylation groups that showed significant differences in clinical outcome and biology: Merlin-intact (MI), immune-enriched (IE), and hypermitotic (HM). MI meningiomas had the best outcomes and were enriched for meningiomas with non-NF2 driver mutations such as *TRAF7*, *AKT1*, and *KLF4*. Merlin expression was found to have pro-apoptotic tumor-suppressor effects in vitro and in vivo leading to increased response to cytotoxic therapies such as RT. IE meningiomas were found to have greater immune cell infiltration of the tumor microenvironment based on DNA methylation, bulk RNAseq, and scRNAseq. These meningiomas had increased expression of *HLA* genes and meningeal lymphatic genes including *LYVE1, CCL21,* and *CD3E*. Lastly, HM meningiomas were enriched for *FOXM1* signaling pathways, leading to increased cell proliferation. Over-expression of *FOXM1* in vitro were found to lead to increased meningioma cell resistance to cytotoxic chemotherapy. However, the majority of HM meningiomas did not have any copy number loss of CDKN2A/B or aberrant activation of the *FOXM1* pathway. Instead, there appeared to be multiple convergent pathways affecting cell cycle in these tumours. Therefore, when tested, CDK4/6 inhibitors, which target a key cell cycle checkpoints, were found to effectively attenuate cell growth of HM meningiomas in vitro [52]. Interestingly, subsequent re-analysis of their own published data by the authors demonstrated that within their HM group of meningiomas, there were two distinct subgroups: one that was characterized by gene expression pathways affecting macromolecule metabolism, and the other with poorer clinical outcomes characterized by enrichment of cell cycling and proliferative pathways. [53]

### Consensus molecular classification for meningiomas

**Fig. 1:**
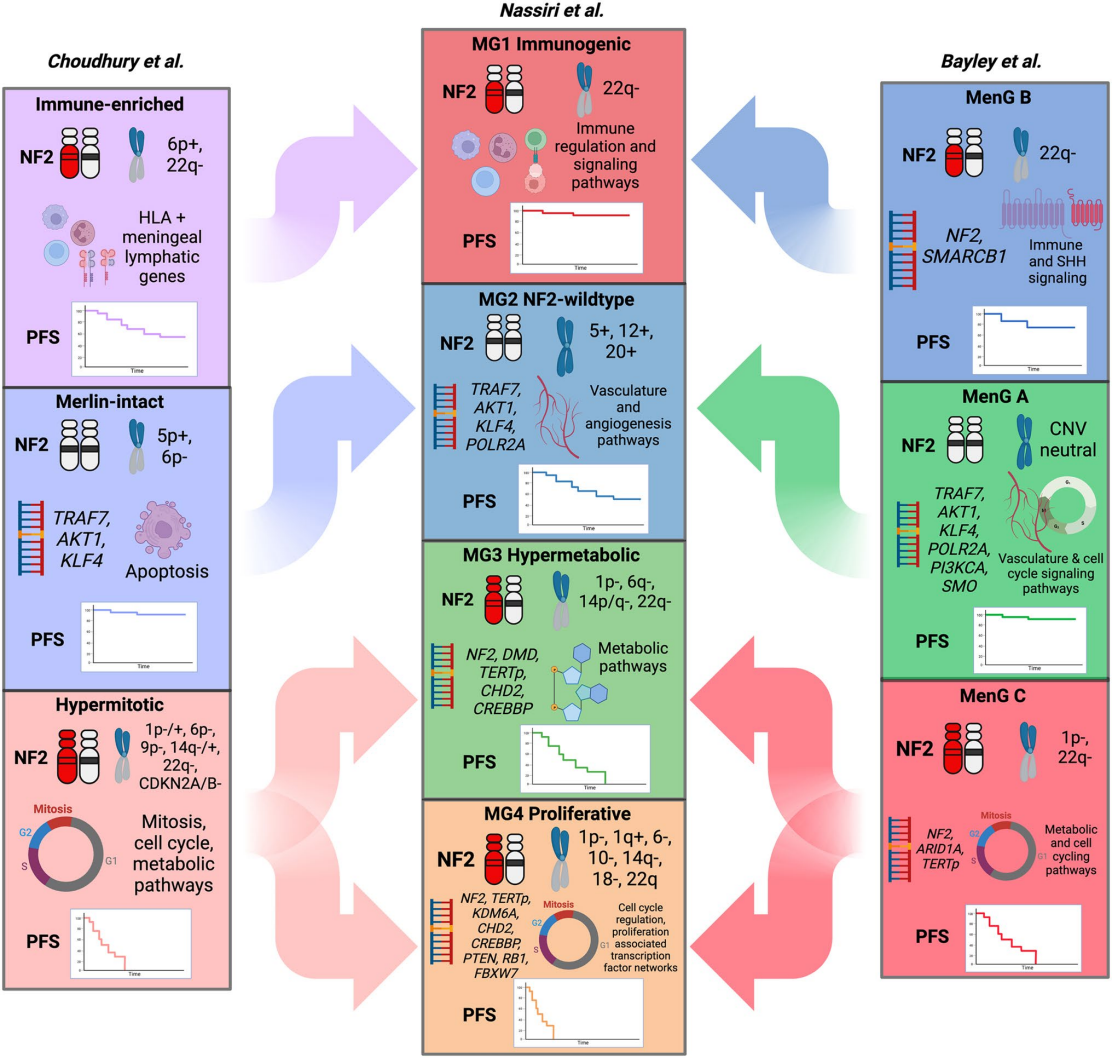
Schematic of how each of these molecular groups derived from recently published multiomics studies likely correlate with one another based on NF2 status, copy number alterations, mutational status, transcriptional pathway, and outcome [schematic of relative progression-free survival (PFS) as defined by each respective study]

While there are differences in the nomenclature of these multiomic molecular groups, there are clear similarities in the biology of specific groups across these three studies and all trump existing WHO and single platform classification (Fig. 1). For example, MG1 (immunogenic) meningiomas from our study logically corresponds to the IE group from Choudhury et al., and MenG B from Bayley et al.; these groups are largely comprised of *NF2*-altered, benign meningiomas enriched for pathways involved in immune signalling and immune cell infiltration of the tumor microenvironment. MG2 or *NF2*-wildtype meningiomas likely belong to the same group as the MI and MenG A groups, comprised of benign-behaving meningiomas with non-*NF2* driver mutations [50–52]. The two subgroups of the HM group from the Choudhury et al. study that were uncovered in re-analysis almost identically match the hypermetabolic (MG3) and proliferative (MG4) groups from our study [53]. The lack of a fourth group in the Bayley et al. study may be partly attributed to the lack of WHO grade 3 and recurrent meningiomas in their cohort, which are generally more aggressive. However, even with the exclusion of these tumors, it is likely that the MenG C group combined with the unclassified meningiomas in their cohort (N = 10; 9%) could be further subdivided into two molecular groups based on the transcriptomic heterogeneity of the MenG C meningiomas in their study [51]. This is further supported by the fact that the MenG classification only dichotomized clinical outcomes into “good” and “poor”, as there were no significant differences in PFS between the MenG A and MenG B tumors. However, it has been demonstrated that there are a group of meningiomas with intermediate outcomes somewhere on the spectrum between the benign behaving meningiomas and the clinically aggressive ones [50, 51, 53]. Therefore, it appears there are likely four distinct groups of meningioma, congruent with what we initially discovered, and supported by reanalysis of published data [50, 53]. However, future efforts should look to harmonize the nomenclature surrounding these groups so that they may be more readily adapted for routine clinical use.

## Future directions

### Pharmacologic treatment of clinically aggressive meningiomas

Currently, the only standard of care therapies for meningiomas are surgery and RT [2]. Patients with meningiomas that are refractory to these modalities contribute to most of the morbidity and mortality of these tumors. However, these recent studies that have comprehensively catalogued the molecular landscape of meningiomas have also uncovered novel therapeutic vulnerabilities that may merit further investigation in future clinical trials. In our study, by mapping Food and Drug Association approved drugs to target genes representative of each MG, we found that vorinostat, a histone de-acetylase inhibitor, could target several key pathways upregulated in the aggressive MG4/proliferative meningiomas [50]. In the Choudhury et al. study, CDK4/6 inhibitors abemaciclib, palbociclib, and ribociclib were found to halt clonogenic growth of established meningioma cell lines (CH157-MN, IOMM-Lee), which were classified as HM group, and IE patient-derived cell lines [52]. Horbinski et al. found that palbociclib decreased tumor cell viability in vitro and improved survival in vivo when combined with RT for targeting p16-deficient (*CDKN2A* deleted) CH157 and IOMM-LEE cells but were ineffective for p16-intact and Rb-deficient meningiomas [54]. Importantly, established in vitro models of meningioma have several limitations, and it remains uncertain how well they recapitulate clinical meningioma samples. Selection of meningioma patients for future trials, particularly in the context of these targeted therapies, should ideally be performed using molecular stratification instead of traditional criteria such WHO grade alone.

### Response to RT in meningiomas

As it stands, RT is generally reserved for the treatment of higher grade (WHO grade 3), incompletely resected (grade 2), or recurrent meningiomas [2]. However, its role for incompletely resected WHO grade 1 meningiomas, totally resected grade 2 meningiomas, and the optimal timing of RT (adjuvant vs. salvage therapy) remain uncertain [55–57]. Furthermore, the efficacy of fractionated RT or stereotactic radiosurgery as primary or first-line treatment of meningiomas that would also be eligible for surgery is an area of active investigation [57–61]. Despite the recent breakthroughs in the molecular subtyping of meningiomas, there are currently no reliable molecular biomarkers of response to RT. Though these recently uncovered molecular groups and other profiling methods such as DNA methylation have been found to be able to robustly predict clinical outcomes following surgery, their ability to similarly predict response following RT remains uncertain [45, 46, 50, 53]. Review of our own unpublished data have shown that a significant proportion of WHO grade 2 and 3 meningiomas will still progress following RT and that there are a paucity of clinical factors that can predict PFS post-RT. Therefore, identification of molecular biomarkers of resistance to RT and the mechanisms through which this occurs are also needed to compliment future targeted therapies for aggressive treatment-refractory meningiomas.

## Conclusion

Though recent studies have independently derived different numbers of molecular or methylation groups, each with their own nomenclature, there appear to be clear similarities in the meningiomas across specific groups from an observational and biological standpoint. However, several additional steps are likely required before these groups can be integrated into routine clinical practice. Firstly, there should be standardization of the number of molecular/methylation groups (4 vs 3), followed by harmonization of the nomenclature surrounding these groups (e.g. “immunogenic” = “immune-enriched”; “merlin-intact” = “NF2-wildtype”, etc.). Secondly, biological validation of these groups using a data driven approach in novel cohorts is required such that any given meningioma can be reliably classified into a consensus molecular group. Lastly, the identification of unique molecular biomarkers or alterations that can be readily identified using a gene panel or IHC (or combination of IHCs) would be a necessity, particularly for centers where routine molecular testing or sequencing may not be readily available. These recent landmark multiomic studies on meningiomas have provided not only the framework necessary for a much-needed paradigm shift in the classification of these tumors but have also produced invaluable genomics data that be used as a resource for future studies moving forward. Only with a complete understanding of the biology of these tumors, will we be able to develop meaningful molecularly targeted therapies for treatment-refractory cases across, and within each molecular group.

## Data Availability

There were no new genomics data generated for this review. Public data availability for the studies cited in this article are available at their respective journal publication sites.
